# Cytology and cell‐block immunohistochemistry of circulating tumour cells

**DOI:** 10.1111/cyt.12770

**Published:** 2019-10-08

**Authors:** Frederick George Mayall, Justin Pepperell, Ian Bodger, Daniel Higbee, Lara Stevanato, Arianna Hustler, Kyra Mhairi Mumford

**Affiliations:** ^1^ Department of Cellular Pathology Musgrove Park Hospital Taunton UK; ^2^ Department of Respiratory Medicine Musgrove Park Hospital Taunton UK; ^3^ Academic Respiratory Unit North Bristol NHS Trust Lung Centre Bristol UK; ^4^ Research and Development Angle PLC Guildford UK

**Keywords:** blood, carcinoma, circulating tumour cells, cytology, immunohistochemistry, megakaryocyte

## Abstract

**Objective:**

The study set out to assess the feasibility of using Parsortix^TM^ circulating tumour cell (CTC) extraction and CytoFoam Disc cell‐block immunohistochemistry to diagnose metastatic carcinoma from blood samples in a National Health Service district general hospital.

**Methods:**

Blood samples were taken from 50 patients with metastatic carcinoma and 50 healthy volunteers and processed, using a previously published method, to extract CTCs and collect them in a cell‐block for routine formalin‐fixed paraffin sectioning and immunohistochemistry. The extracted cells were compared with the patients’ routine diagnostic samples.

**Results:**

The samples from the 50 carcinoma patients showed cytokeratin‐positive cells in 19 cases. In eight of these, the cytokeratin‐positive cells had a similar immunoprofile to the carcinoma in the conventional biopsy or cytology specimen. Some carcinoma patients also had circulating cytokeratin‐positive cells that were probably benign epithelial cells and circulating megakaryocytes. Both of these types of cells were also found in healthy volunteers. Processing and initial examination could be completed in 2 days. The full processing cost was approximately £316 per case.

**Conclusions:**

CTCs could be extracted from the blood of some patients with metastatic carcinoma and formed into a formalin‐fixed cell‐block for routine paraffin processing and immunohistochemistry. The specificity of this approach is constrained by the observation that some patients with metastatic carcinoma had circulating cytokeratin‐positive cells that were probably benign, and these were also found in healthy volunteers. Circulating megakaryocytes were present in carcinoma patients and healthy volunteers.

## Abstract 2

Circulating tumour cells were extracted from the blood of patients with metastatic carcinoma and formed into formalin fixed cell blocks for routine paraffin processing followed by immunohistochemistry that showed a match with the patient's carcinoma. Some patients with metastatic carcinoma had circulating cytokeratin positive cells that were probably benign cells and similar cells were also found in healthy volunteers.

## 1 INTRODUCTION

There is currently a lot of interest in circulating tumour cells (CTCs), and their potential in liquid‐based tumour diagnosis. In this context, a CTC usually refers to a circulating cell from a solid tumour, such as a carcinoma, melanoma or sarcoma, and haematological malignancies are excluded. CTCs are not a recent discovery. There is a description of tumour cells in post‐mortem blood from 1869.^1^ They were found in a man who had approximately 30 subcutaneous tumours. It is quite likely that this patient actually had a haemato‐lymphoid malignancy with a leukaemic component, as the malignant cells in the blood appear to have been much more numerous than our modern experience of CTCs. There is a more reliable and more detailed description of CTCs in living patients with carcinomas, melanomas and sarcomas from the 1950s, along with a description of other rare benign circulating cells, including macrophages and megakaryocytes, that can mimic CTCs.^2^ Most recent studies have concentrated on extracting and analysing neoplastic circulating epithelial cells, with the aim of developing techniques to diagnose and characterise carcinomas. Non‐neoplastic circulating epithelial cells have received relatively little attention, partly because of their rarity and the consequent difficulty in studying them. However, there are reports of non‐neoplastic circulating cytokeratin‐positive cells (NCCCs) in patients with benign diseases, including prostatitis and Crohn's disease, and after benign breast surgery.^3^, ^4^, ^5^, ^6^ It has been thought that NCCCs are almost never detectable in healthy subjects.

Recently, we described a method for preparing a cell‐block from a very sparsely cellular extract of CTCs and other rare circulating cells, and examining the cells with routine diagnostic methods including formalin‐fixed paraffin sections and immunohistochemistry.^7^

The study described here sets out to assess the feasibility of using this method to diagnose metastatic carcinoma in the setting of a National Health Service district general hospital in the UK, mainly using methods that are in routine use in diagnostic cellular pathology laboratories. We focused on the type of rare circulating cells recovered, quality of preservation, concordance with the associated diagnostic sample, turn‐around times and cost.

## 2 METHOD

We recruited 50 patients with metastatic carcinoma together with 50 healthy volunteers, following a protocol approved by an ethics committee. Blood was drawn from each of these subjects. For the first 15 carcinoma patients, this was a volume of 10 mL collected in a single EDTA Vacutainer tube, but for all other carcinoma patients this was increased to 20 mL, collected in two tubes, and also 20 mL for all of the 50 healthy volunteers. The reason for the change from 10 mL to 20 mL was the discovery of probable NCCCs and megakaryocytes in the early samples and it was decided that an additional 10 mL of blood should be taken for Parsortix^TM^ extraction and cytological examination. These samples were processed through a Parsortix™ PR1 device as quickly as possible, either the same day or the next day. The manufacturer's PX2_ANG_002_SH_90 protocol was used. Two of these devices were available, so that two tubes of blood could be extracted in parallel. The extract from one blood tube was used to form a cell‐block, and the extract from the other tube was used for cytology.

The processing through the Parsortix™ device and the formation of a cell‐block has been described in detail previously.^7^ Briefly, as the blood was processed through the Parsortix™ device, cells larger than approximately 6 μm were retained by the filter cassette. These were then recovered by a phosphate buffered saline back wash of the cassette, yielding 90 μL extract. Next, a 40 μL droplet of plasma was placed at the centre of a clean glass microscope slide. The plasma was derived from the patient's own blood sample. A 12‐mm Cytofoam Disc was placed on top of the droplet on the slide and the 90 μL phosphate buffered saline extract containing the recovered cells was added to the centre of the top face of the disc. A further 40 μL of plasma was then added. The surface of the disc was then prodded with a micropipette tip 30 times to encourage mixing of the fluids. A 500‐mL plastic histology specimen container was then prepared with a folded paper towel across its floor that was soaked with 5 mL of neutral buffered formalin. The glass slide was then placed flat on top of this with the Cytofoam Disc uppermost. The container's lid was closed and left at room temperature for 24 hours for the sample to fix in the formalin vapour. The slide was then removed from the container and the Cytofoam Disc prised from the surface of the slide with the edge of a scalpel. The disc was then wrapped in tissue paper and paraffin processed as for a biopsy specimen. This method was designed to minimise the loss of cells during processing with recovery of the cells directly into the CytoFoam Disc cell‐block matrix.

Following paraffin processing, five cell‐block serial sections were placed on one slide, along with suitable positive and negative controls, and immunostained for MNF116 (a broad‐spectrum cytokeratin antibody).^7^ If this slide showed no positive cells then no further immunohistochemistry was undertaken. If MNF116‐positive cells were found, additional immunohistochemistry was performed. If the patient was male, immunohistochemistry for CK7, CK20, TTF1, CDX2, PSMA and PAX8 was performed, and, if female, then for CK7, CK20, TTF1, CDX2, PAX8, oestrogen receptor (ER), WT1 and mammoglobin. In a few healthy volunteer cases, CD31 immunohistochemistry was used to confirm that atypical cytokeratin negative cells were megakaryocytes.

The Parsortix extraction for cytological examination was performed on the second 10 mL EDTA tube of blood, in the same way as for cell‐block creation except that the extract was recovered directly onto a glass slide as 3 or 4 small droplets (together 90 μL). The slide was air‐dried without being smeared, and then methanol fixed and stained with Speedy‐Diff following the manufacturer's instructions. The slide was then examined for the presence of CTCs, NCCCs and megakaryocytes.

The microscopic appearances and immunoprofile of the extracted circulating cells in the cell‐block were compared with the appearances and immunoprofile of the carcinoma in the related conventional biopsy or cytology sample.

## 3 RESULTS

### 3.1 Patients

The patients included 19 males and 31 females with a mean age of 70 years (Table 1). All had metastatic carcinoma. These included 23 cases with metastatic lung carcinoma, five with ovarian carcinoma, four with endometrial carcinoma, four with breast carcinoma, one with colorectal, one with prostatic, one with large bowel carcinoma, one with bladder carcinoma and six with carcinoma of unknown origin.

**Table 1:** Cases with metastatic carcinoma, and circulating cytokeratin‐positive cells

Case	Age	Sex	Cytology & IHC	Biopsy or cytology diagnosis: IHC positive	CTC result
1	69	F	MNF116, CK7, TTF1 (Figure 1)	Poorly differentiated adenocarcinoma of lung: CK7, TTF1 & ER (weak)	Probably neoplastic and indicates site of origin
4	68	M	MNF116, PAX8 & CK7	Metastatic carcinoma, probably colorectal: CK20, CDX2	Uncertain cell type
9	75	F	MNF116, CK7, CK20 & WT1 (Figure 3)	Clear cell carcinoma of kidney: MNF116, PAX‐8, vimentin & CD10	Uncertain cell type
11	70	F	MNF116 & CK7	Adenocarcinoma of lung: CK7, BerEP4 & TTF1	Uncertain cell type
18	77	F	MNF116 & CK7	Metastatic lung carcinoma: CK7	Probably neoplastic
22	55	F	MNF116, CK7 & ER	Invasive ductal carcinoma of breast	Probably neoplastic
26	60	M	MNF116 & CK7	Metastatic adenocarcinoma of lung in liver: CK7 and TTF1	Probably neoplastic
27	79	F	MNF116 & WT1	Grade 3 endometrioid adenocarcinoma of endometrium	Uncertain cell type
28	49	F	MNF116 & ER	Invasive ductal carcinoma breast, grade 3: ER & Her2	Uncertain cell type
30	75	F	MNF116, CK7 & CK20	Serous ovarian carcinoma: Ber‐EP4, CK7, WT1, ER & p53	Uncertain cell type
31	68	M	MNF116	Metastatic carcinoma probably squamous carcinoma: p63, CAM5.2 & CK7	Uncertain cell type
32	66	M	MNF116 & CK20	Adenocarcinoma of lung: CK7, TTF1, BerEp4 & CEA	Uncertain cell type
35	75	F	MNF116, CK7, WT1 & CK20	Adenocarcinoma: no IHC	Uncertain cell type
36	62	M	MNF116, CK7 and TTF1 (Figure 2)	Small cell carcinoma: MNF116, TTF‐1, CK7, CD56 & synaptophysin	Probably neoplastic and indicates site of origin
37	61	M	MNF116, CK7 & CK20	Squamous carcinoma of lung: no IHC	Uncertain cell type
41	77	M	MNF116 & CK7	Adenocarcinoma of lung: BerEP4, CK7 and TTF‐1	Uncertain cell type
47	79	F	MNF116, CK7	Adenocarcinoma, perhaps mucinous ovarian: CK7, CK20 & CDX2	Probably neoplastic
48	73	F	MNF116, CK7 & TTF1	Adenocarcinoma of lung: TTF1	Probably neoplastic and indicates site of origin
50	77	F	MNF116, CK7, ER	Metastatic breast carcinoma: MNF116, CK7, ER & GATA3.	Probably neoplastic

Note

In some cases these circulating cells were probably neoplastic, matching the features of the diagnostic biopsy or cytology specimen, sometimes with an immunoprofile that suggested a likely site of origin of the carcinoma. For the other cases, it was not possible to show that the circulating cells were similar to the carcinoma, with some showing features markedly different to those of the carcinoma, suggesting that they may not be neoplastic.

ER, oestrogen receptor; IHC, immunohistochemistry; CTC, circulating tumour cell.

### 3.2 Cell blocks

The samples usually arrived in the laboratory between 10 and 20 minutes after being taken. Extraction appeared to be quicker if started immediately after the sample had been taken, and conversely samples that were refrigerated overnight proceeded less quickly. For the latter, when the extracted sample was examined there were more prominent aggregates of platelets and white blood cells and we believe that these may have interfered with the movement of the blood through the Parsortix^TM^ filter. Samples usually took 3‐5 hours to be processed on the device, but there was some variability, the quickest taking 1 hour 28 minutes and the slowest 5 hours 50 minutes. The initial immunohistochemistry for MNF116 immunohistochemistry was usually available between 5 and 7 days from the time the sample was taken, with some variability, the shortest time being 2 days. For those samples that had immunohistochemistry for male or female carcinoma panels, the time from the sample being taken to the carcinoma panel being available for examination was approximately 10‐12 days, although in some cases it was available in 6 days. The turnaround times in our study were constrained by the prioritisation of clinical diagnostic work over the requirements of this research study. If the testing had the same priority as routine diagnostic work, then we believe that the minimum turnaround times described above would have been achievable for most cases. The mean cost per case was approximately £316. This compares well with the cost of an ultrasound‐ or computed tomography‐guided core biopsy which would add at least £500 to the price. Image guided biopsies are more risky for the patient, require repeat hospital attendance and often take several days to arrange.

### 3.3 Cytology

As described in the Methods, cytology was performed from Case 16 onwards in an attempt to examine the morphology of the extracted cells and thus determine if they were likely to be benign or neoplastic. The preparation time was essentially the same as the transport time and extraction time as for cell‐blocks. Drying of the glass slide and staining and coverslipping only took a few extra minutes. The cost is estimated at approximately £20.

### 3.4 Identification of cells in cancer patients

**Figure 1:**
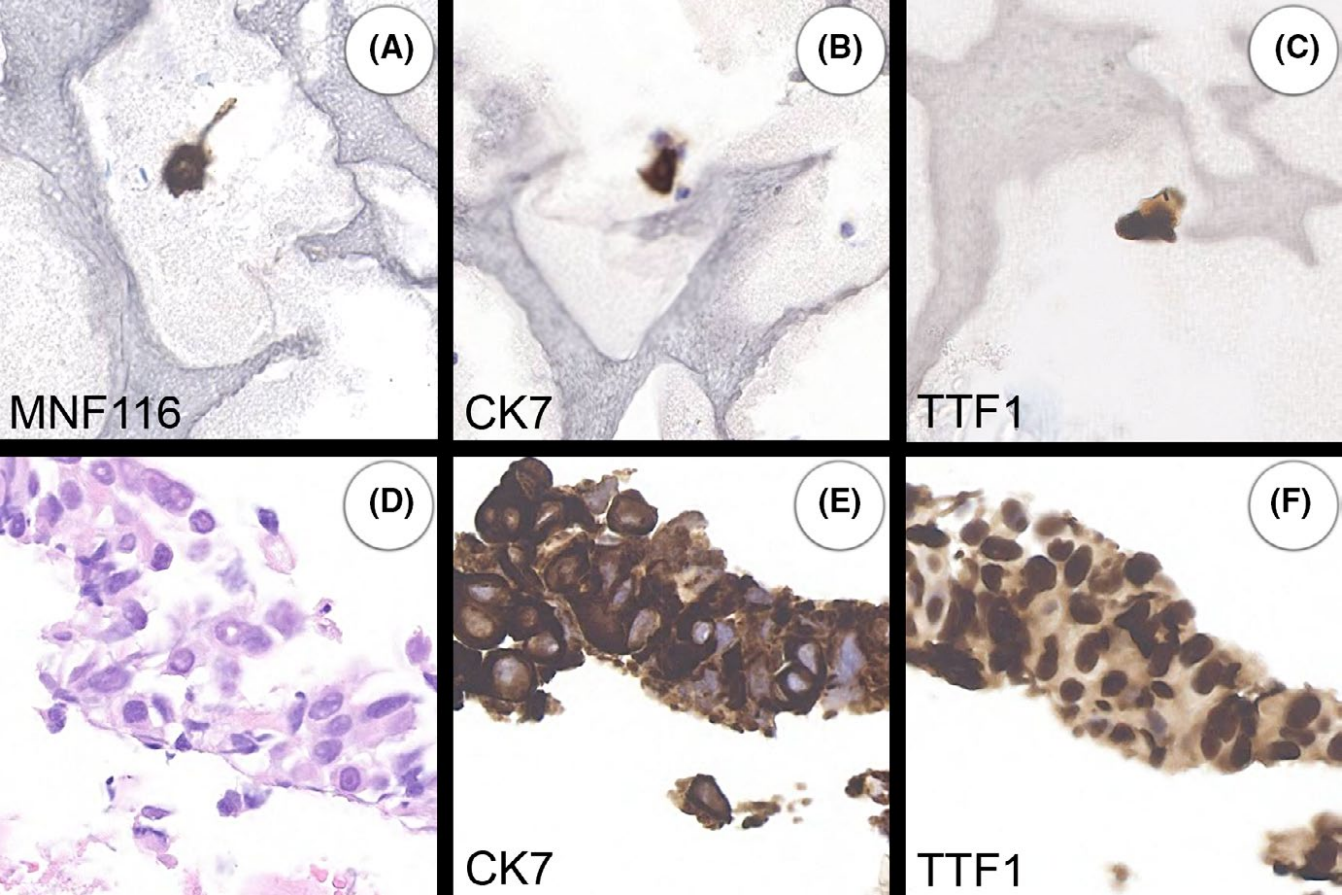
Case 1. A 69‐year‐old woman with poorly differentiated lung carcinoma and circulating cells (A‐C) that were similar morphologically and immunophenotypically to those in the biopsy (D‐F). All images are at the same magnification

**Figure 2:**
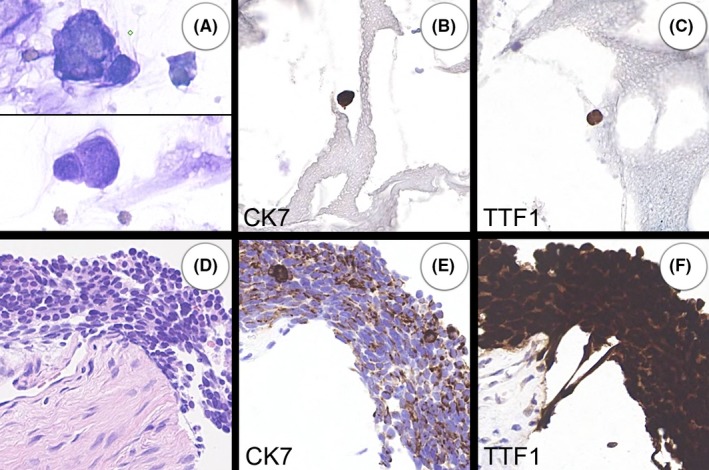
Case 36. A 62‐year‐old man with small cell lung carcinoma. Poorly preserved atypical circulating cells could be seen on cytology (A). The circulating cells (A‐C) are morphologically and immunophenotypically similar to those in the biopsy (D‐F). All images are at the same magnification except for the cytology (A)

The results are set out in Table 1. The samples from the 50 carcinoma patients showed MNF116‐positive cells in 19 cases. In eight of these, the cytokeratin‐positive cells were shown to have a similar immunoprofile to the carcinoma in the related biopsy or cytology specimen (Figures 1 and 2).

**Figure 3:**
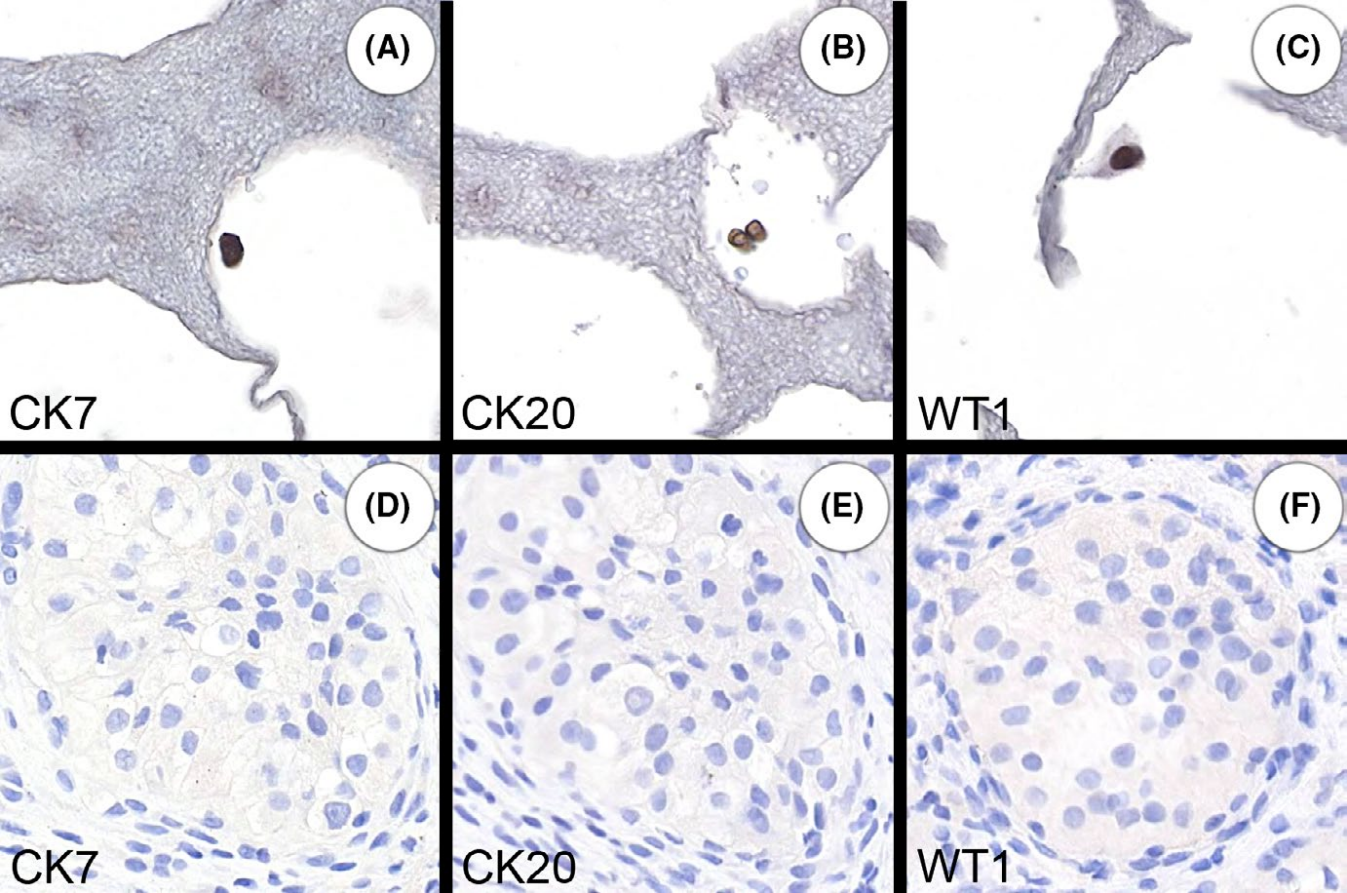
Case 9. A 75‐year‐old woman with clear cell carcinoma of the kidney. The circulating cytokeratin‐positive cells (A‐C) do not resemble those of the carcinoma (D‐F) morphologically or immunophenotypically

For the remaining 11 cases with cytokeratin‐positive cells, it was not possible to be certain that these cells were the same as those in the patient's carcinoma. In some cases, this was because there were only scanty cells and this constrained the reliability of the interpretation of the immunostains. However, for some cases the circulating cytokeratin‐positive cells appeared to be distinctly different from the primary carcinoma. Their immunoprofile was inconsistent with the carcinoma from the diagnostic specimen, and their morphology was also different. In some cases, the morphology suggested that the cells were probably benign epithelial cells, as they had small regular nuclei. In Case 9, the cell‐block showed MNF116‐positive cells that were also positive for CK7, CK20 and WT1 (Figure 3). The latter three markers were negative in the patient's clear cell renal carcinoma, and the morphology was different to that of the renal carcinoma. This suggests that the circulating cells were NCCCs, but it is also notable that there is no normal organ that has cells that co‐express these markers, further suggesting that there may have been two different types of NCCCs. There also appears to be a mismatch between circulating cells and the carcinoma for cases 4, 30 and 32. For Case 37, the patient had squamous carcinoma, diagnosed without immunohistochemistry, but the circulating cells were positive for CK7 and CK20 which is not typical of squamous carcinoma. It is possible that in some cases a mixture of neoplastic and non‐neoplastic cells was obtained.

**Figure 4:**
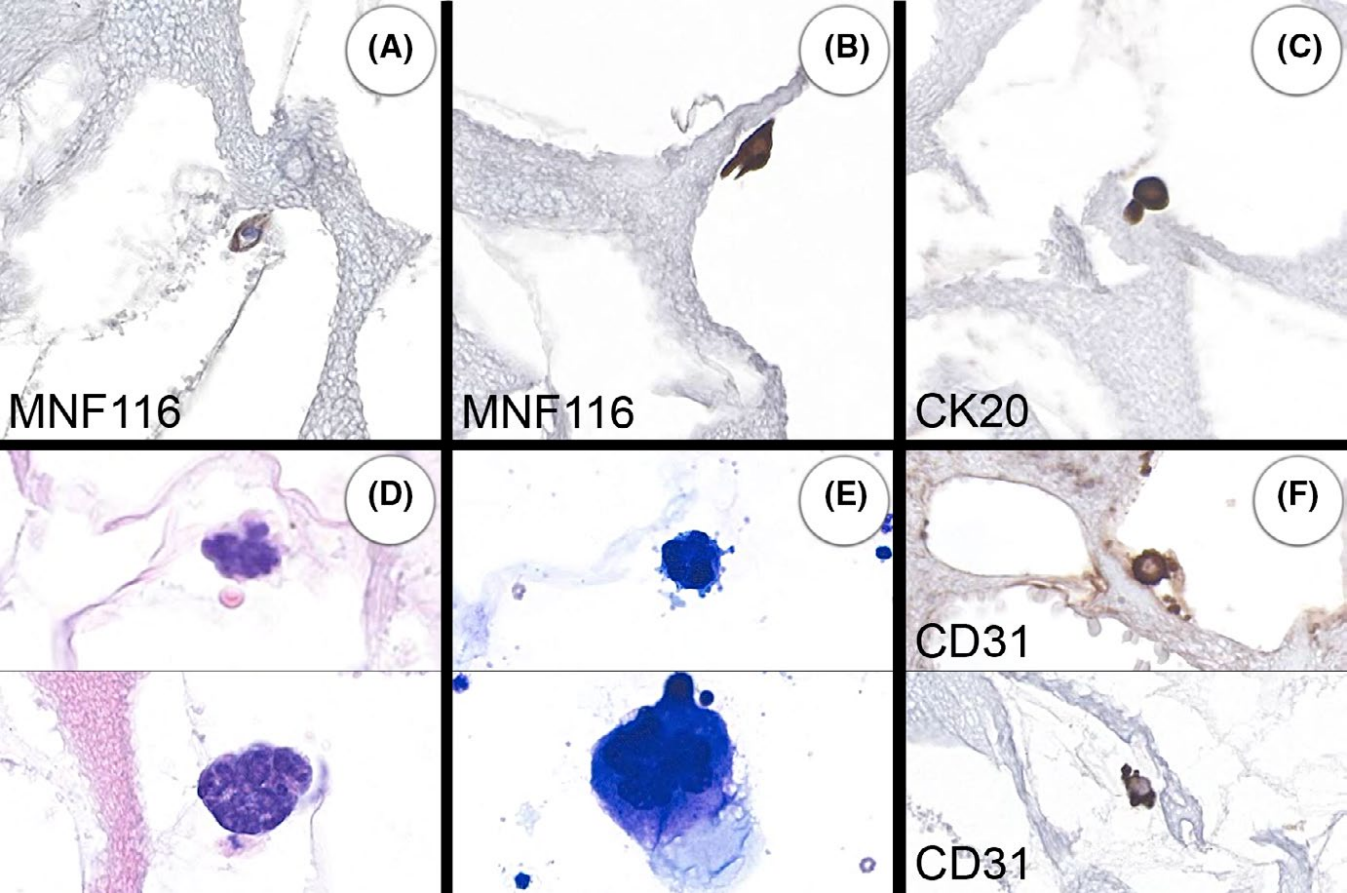
(A‐C) Cytokeratin‐positive cells in normal healthy volunteers. These have small benign‐looking nuclei. Image B includes a cell with a sharply angulated cytoplasmic profile, suggestive of squamous differentiation. (D‐F) Megakaryocytes that show substantial variation in size. Many had stripped cytoplasm (D). The CD31 stain highlights some micro‐megakaryocytes that could have been mistaken as circulating tumour cells on morphology. All images are at the same magnification except for the cytology (E)

Cytology was performed on 35 of the metastatic carcinoma cases and 14 of these showed megakaryocytes. Others had ambiguous cells for which it was not possible to confidently distinguish between CTCs, NCCCs, degenerate megakaryocytes and micro‐megakaryocytes. This was in part due to the prominent artefact that many of the cells showed, particularly the stripping of the cytoplasm. Occasional cells were well preserved (Figure 4D‐F).

### 3.5 Circulating cytokeratin‐positive cells in healthy volunteers

Circulating MNF116‐positive cells were found in the blood of 12 of the 50 healthy volunteer samples (Figure 4A‐C). Of these 12, there were six that also showed positivity for CK7 and one that was CK7 negative but showed positivity for CK20. The remaining five were only positive for MNF116. One of these five seemed to show morphological evidence of squamous differentiation (Figure 4B).

### 3.6 Circulating megakaryocytes in healthy volunteers

Circulating megakaryocytes were found in 8 of the 50 healthy volunteer samples (Figure 4D‐F). These were detected by morphological examination and therefore micro‐megakaryocytes (Figure 4F) would have tended not to be recognised as megakaryocytes.

## 4 DISCUSSION

The study set out to assess the feasibility of using this method to diagnose metastatic carcinoma. While cost and turnaround times are potentially an improvement on alternatives such as an ultrasound and computed tomography‐guided core biopsy, the sensitivity was suboptimal. Many of the patients with metastatic carcinoma did not have detectable CTCs using this method. In addition, the specificity of this approach is constrained by the observation that some patients with metastatic carcinoma had circulating cytokeratin‐positive cells that were probably benign NCCCs and these were also found in healthy volunteers. It might be possible to increase the volume of the blood sample and this is likely to increase the yield of circulating epithelial cells, but in order to increase the volume substantially, one would probably have to resort to a recirculation method such as diagnostic leukapheresis. In this method, up to 2.5 L of blood may be sampled^8^ and returned to the patient. However, such a specialist technique would increase the complexity and cost of the method described above, and so make it a less compelling alternative to routine biopsy methods. In some patients with clinically suspected metastatic malignancy, there is no obvious target mass for biopsy on imaging and so leukapheresis might be a useful strategy. Another approach would be to screen all carcinoma patients to find those that had CTCs, and for those patients use repeat testing to monitor treatment and progression.

The study has provided some unexpected new insights into rare circulating cells. Firstly, in patients with metastatic carcinoma it appears that some circulating cytokeratin‐positive cells were probably benign background circulating epithelial cells, and these were also found in healthy volunteers. Secondly, circulating megakaryocytes were commonly seen in patients with metastatic carcinoma, and these were also seen, albeit less frequently, in healthy volunteers.

As mentioned above, we have known that NCCCs can be found in some diseases, but most studies have found them to be absent in healthy volunteers. One study using CellSearch^®^ (an immuno‐magnetic recovery method) found one circulating epithelial cell per 7.5 mL of blood in 5.5% of 145 healthy women,^9^ but no sample had more than one cell. Although our method did not attempt to count the number of cells exactly, in one healthy volunteer sample, there were up to three cytokeratin‐positive cells on a single immuno‐stained slide bearing five cell‐block paraffin sections, each 3‐μm thick.

Recent studies have shown that in some patients with cancer there are CTCs with frequent gene copy number alterations but there are sometimes also a minority of circulating epithelial cells with few if any gene copy number alterations.^10^ The latter may be background benign epithelial cells.

Some studies have found circulating epithelial cells in carcinoma patients that were discordant with the immunophenotype of the carcinoma. In one study,^11^ ER‐negative circulating epithelial cells were found in a patient with ER‐positive breast carcinoma. In this previous study, CTCs were detected in blood of 16 from 35 patients with ER‐positive breast carcinomas, with a median of 3 CTCs/7.5 mL. In total, ER‐negative CTCs were detected in 11/16 (69%) of the CTC‐positive cases, including blood samples with only ER‐negative CTCs (19%) and samples with both ER‐positive and ER‐negative CTCs (50%). No correlation was found between the intensity and/or percentage of ER staining in the primary tumour with the number and ER status of CTCs of the same patient. The authors of this study suggest that the results could be evidence of heterogeneity of the tumour cells, but an alternative explanation could be that some of the cells were not CTCs, but are benign circulating epithelial cells.

Another study of metastatic breast carcinoma patients^12^ detected and characterised CTCs in 38.5% of the patients with a total of 92 CTCs. It could demonstrate that at least 69.6% of the CTCs exhibit an ERα and/or ErbB2 status different from the status of the primary tumour and that the CTCs from only 30% of the patients had no change of receptor status. However, CTC clusters observed in this study for four patients always exhibited the same receptor profiling within one cluster. Again, it is possible that the explanation could be related to the presence of benign cytokeratin‐positive circulating cells.

A study of patients with pancreatic tumours^13^ found that no circulating epithelioid cells (cells resembling epithelial cells on cytology) were identified among nine normal healthy controls. Of the 115 patients with circulating epithelioid cells, 25 had nonmalignant disease and 90 had malignancy. There were no significant differences in any of the cytological criteria noted between groups divided by benign vs malignant, neoplastic vs non‐neoplastic, or pancreatic ductal adenocarcinoma vs neuroendocrine tumour. The authors concluded that care must be taken not to overinterpret cells identified by cytomorphology as indicative of circulating tumour cells of pancreatic cancer.

In our study, most of the NCCCs were positive for CK7, indicating a likely origin from the epithelium of the upper gastro‐intestinal tract, female genital tract, lung and breast. There is also the possibility that they could be contaminants from skin adnexal glands at the time of the sample being taken. It is assumed that NCCCs are epithelial, but there are other alternative cytokeratin‐positive cells including mesothelial cells and synovial cells. Both of these are MNF116 and CK7 positive. However, no WT1‐positive NCCCs were found, suggesting that a mesothelial origin is unlikely.

It is well known that megakaryocytes can be seen in the peripheral blood of patients with myeloproliferative disorders. However, it is less well appreciated that they can also be found in the peripheral blood of patients with carcinoma and non‐neoplastic disorders, and also healthy subjects.^14^, ^15^ They seem to be more common in patients with advanced malignancy than early stage malignancy and benign conditions. Megakaryocytes are commonly seen in pulmonary capillaries.^16^ It is possible that other researchers have found megakaryocytes while looking for CTCs but have not recognised them. Images of megakaryocyte‐like cells have been published in at least one previous paper on CTCs.^13^ In the current study, we also found that megakaryocytes were more common in patients with carcinoma than in healthy volunteers. Many of the megakaryocytes appeared to have lost their cytoplasm. While this could be due to the physical trauma of being trapped by the Parsortix^TM^ filter, similar features were seen when enzymatic streptolysin O haemolysis was used to separate the megakaryocytes from other blood cells.^14^

In summary, this study shows that CTCs can be extracted from the blood of some patients with metastatic carcinoma, and that routine formalin‐fixed cell‐block immunohistochemistry can be used to demonstrate that these CTCs have features similar to those of the tumour biopsy. Processing and examination can be done in 2 days, if prioritised, in a National Health Service district general hospital laboratory mainly using standard techniques developed for routine biopsy specimens. The processing cost is approximately £316 per case. This study also indicates that benign cytokeratin‐positive cells and megakaryocytes are present in the blood of patients with metastatic carcinoma and in healthy volunteers. A follow‐on study is planned that will attempt to capture CTCs in patients with large cell lymphoma.

The Parsortix CTC extraction method described above is not approved for clinical use.

## CONFLICT OF INTEREST

Taunton and Somerset NHS Foundation Trust received a grant from ANGLE Europe Limited to support this research and is the employer of F. Mayall and J. Pepperell, and the former employer of D. Higbee. ANGLE Europe Limited is the employer of L. Stevanato, A. Hustler and K. Mumford.

## AUTHOR CONTRIBUTIONS

F. Mayall—Study design, regulatory approval, method development, administration and principle author. J. Pepperell—Study design, regulatory approval and administration. D. Higbee—Administration and patient recruitment. I. Bodger, Lara Stevanato and Arianna Hustler—Method development and administration. K. Mumford—Study design and method development.

5

## Data Availability

The data that support the findings of this study are available from the corresponding author upon reasonable request.
